# One Health in hospital settings: the role of water as an invisible reservoir of opportunistic pathogens

**DOI:** 10.1007/s42770-026-02078-5

**Published:** 2026-09-04

**Authors:** Emilly Brito Ferreira, Jean Lopes da Silva, Monica de Souza Ferreira de Mattos, Hilton Vizi Martinez, Eduardo Jorge Pilau, Maria Cristina Bronharo Tognim, Fabrícia Gimenes, Miguel Machinski Junior, Benício Alves de Abreu Filho

**Affiliations:** 1 State University of Maringá, Post-Graduate Program in Food Science. Av. Colombo, 5.790, CEP 87020-900, Jd. Universitário, Maringá, Paraná Brazil; 2 Department of Basic Health Sciences. Av. Colombo, State University of Maringá, 5.790, CEP 87020-900, Jd. Universitário, Maringá, Paraná Brazil; 3 Maringá University Hospital, State University of Maringá. Av. Mandacaru, 1.590, CEP 87083-240, Pq. das Laranjeiras, Maringá, Paraná Brazil; 4 Department of Chemistry. Av. Colombo, State University of Maringá, 5.790, CEP 87020-900, Jd. Universitário, Maringá, Paraná Brazil

**Keywords:** Hospital water, One Health, Infection control, Surveillance, *Pseudomonas aeruginosa*

## Abstract

**Objectives:**

To evaluate the microbiological quality of a hospital's treated water distribution system, characterize opportunistic bacterial isolates and their antimicrobial susceptibility profiles, investigate the genetic relatedness between environmental and clinical isolates from a One Health perspective.

**Study design:**

Prospective observational study.

**Methods:**

A total of 352 water samples were collected over six months from strategic locations within a public teaching hospital. Samples were analyzed for total coliforms, *Escherichia coli*, heterotrophic bacteria (HB), bacterial isolation and identification, antimicrobial susceptibility testing, and ERIC-PCR genotyping was applied to *Pseudomonas aeruginosa* isolates to analyse their similarity with clinical isolates. Statistical analysis used the chi-square test of independence (χ^2^).

**Results:**

Despite legal compliance for coliforms, elevated HB counts were found in 17% of water tank samples and 12% of ICU samples. Pathogens such as *Pseudomonas aeruginosa*, *Stenotrophomonas maltophilia*, and *Enterobacter* spp. were isolated. *Enterobacter* spp. isolates exhibited resistance to multiple β-lactams. Genotypic analysis did not reveal a direct clonal relationship between *P. aeruginosa* isolates from water and NDM-producing *P. aeruginosa* isolates recovered from hospitalized patients.

**Conclusions:**

Although the hospital water distribution system complied with current microbiological potability standards, opportunistic bacteria were detected in localized areas of the network. The absence of clonal relatedness between environmental and clinical *P. aeruginosa* isolates suggests that the water system was not an active source of dissemination during the study period. These findings support the value of routine environmental surveillance as part of infection prevention strategies within a One Health framework.

**Supplementary Information:**

The online version contains supplementary material available at https://doi.org/10.1007/s42770-026-02078-5.

## Introduction

Water security is a critical component in healthcare settings, where water supply systems are recognized as potential breeding grounds for pathogenic microorganisms [1]. In these systems, biofilms can develop, protecting opportunistic pathogens such as *Acinetobacter* spp., *Legionella* spp., and *Pseudomonas* spp. from disinfectant and increasing the risk of Healthcare-Associated Infections (HAIs). This challenge is further compounded by the intensive use of antimicrobials in healthcare settings, which promotes the emergence and persistence multidrug-resistant bacteria and contributes to the dissemination of antimicrobial resistance genes [2–5]. This scenario highlights the close interconnection between human and environmental health, reinforcing the importance of the One Health approach for understanding and mitigating pathogen dissemination across clinical and environmental compartments [6].

The epidemiological relevance of hospital water systems has been demonstrated in several healthcare-associated outbreaks. Water sources have been implicated in the transmission of carbapenemase-producing *Enterobacteriaceae* in intensive care units (ICUs) in South Korea [7], while *Legionella pneumophila* was detected in hospital wards in Italy [8]. Furthermore, a study in French ICUs demonstrated that almost half of the cases of *Pseudomonas aeruginosa* infections in patients were related to external sources, such as tap water, highlighting the importance of environmental surveillance and preventive strategies to reduce healthcare-associated infections [9].

Although water quality monitoring is routinely performed, the microbiota that colonizes hospital internal distribution networks remains understudied [4]. In Brazil, Ordinance GM/MS N^o^. 888/2021 establishes microbiological potability standards based on the absence of coliforms, and the counting of heterotrophic bacteria (HB) is not mandatory [10]. Nevertheless, HB analysis serves as a valuable tool for assessing the integrity of the distribution system and the potential risk of biofilm formation, complementing surveillance efforts and the understanding of water quality at critical points of use [11, 12].

This study was conducted at the Regional University Hospital of Maringá (HURM), a reference public teaching hospital in southern Brazil [13]. Given its operational nature as a high-complexity training center with intense antimicrobial pressures and high patient turnover, its internal water system represents a critical model for investigating potential microbiological reservoirs. The choice of this location was reinforced by a pre-existing collaboration with the Water, Environmental, and Food Microbiology Laboratory of the State University of Maringá (UEM). Preliminary data indicated the recurrence of bacterial profiles in the hospital's water, prompting an in-depth investigation. To our knowledge, this is the first study to characterize the bacterial community and antimicrobial resistance profiles within the treated water distribution network of this institution.

Therefore, this study aimed to: evaluate the microbiological quality of the water by counting heterotrophic bacteria and analyzing the presence/absence of total coliforms and *Escherichia coli*; characterize the planktonic prokaryotic communities with pathogenic potential and their resistance profiles; and analyze the genetic similarity, using the Enterobacterial Repetitive Intergenic Consensus Polymerase Chain Reaction (ERIC-PCR) technique, between *P. aeruginosa* isolates from the water and New Delhi Metallo-beta-lactamase (NDM) recovered from hospitalized patients, which were previously characterized in a study carried out in the same semester as the collections used in this study [14]. These results aim to contribute to the development of more effective infection control protocols, recognizing aquatic environments as potential sources of risk from the perspective of One Health.

## Methods

### Study site, institutional infrastructure, and sampling design

The study was conducted through environmental microbiological surveillance at the Regional University Hospital of Maringá (HURM), located in Maringá, Paraná, Brazil. HURM is a strategic reference teaching hospital for medium and high-complexity medical care, serving exclusively through the Unified Health System (SUS) a regional macro-population of approximately 2 million inhabitants. The facility operates with 201 inpatient beds across 46 clinical specialties, rendering an environment characterized by high patient turnover and intense antimicrobial baseline usage [13].

The hospital is supplied exclusively by the municipal public water distribution system (Sanepar, Paraná, Brazil), with no alternative groundwater or artesian well sources. The internal water distribution network is approximately 35 years old and consists predominantly of polyvinyl chloride (PVC) piping, with metallic components restricted to the outlet valves of the main reservoirs. Water storage structures undergo professional cleaning and chemical disinfection every six months by a licensed company. The water storage system comprises three primary 3,000-L reservoirs supplying ten secondary 1,000-L water tanks distributed throughout the hospital.

A total of 352 water samples were collected between May and October 2024, in collaboration with the Water, Environmental, and Food Microbiology Laboratory at UEM. Sampling was conducted in standardized monthly intervals across the six-month tracking period. The monthly distribution, of samples is presented in Supplementary Table S1.

Sampling points were selected based on healthcare criticality and risk exposure for immunocompromised patients. For the mandatory total coliform and *E. coli* testing, 253 single-core samples were gathered across 22 locations, including outpatient clinics, intensive care units (ICUs), surgical blocks, and storage reservoirs. Additionally, 99 samples from water tanks and ICU taps were analyzed for heterotrophic bacteria (HB), as these sites represent critical points within the hospital water distribution system.

All samples were collected by trained professionals following the standardized procedures of the Standard Methods for the Examination of Water and Wastewater [15]. Samples were collected from active usage points after a standardized 2-to-3 min flush protocol to eliminate transient point-of-use stagnation variables, being gathered as strategic single core volumes, which prioritized maximum spatial screening across distinct sectors. A volume of 100 mL of water from each point was stored in sterile bottles containing 10 mg of sodium thiosulfate to neutralize residual chlorine, stored under refrigeration, and transported to the laboratory within a maximum time of two hours. While a six-month monitoring window faces inherent limitations in capturing broad macro-seasonal variations, this continuous surveillance period serves as a robust descriptive baseline to investigate immediate bacterial persistence and recurrence profiles within these high-risk hospital niches.

### Microbiological analysis of water

Total coliforms and *E. coli* were detected using the chromogenic substrate technique, using the commercial Colisure® kit (IDEXX Laboratories Inc., USA). The contents of one substrate sachet were added to every 100 mL of water sample and incubated at 35 ± 0.5 °C for 24 h. A color change to magenta indicated a positive result for total coliforms. Positive samples were then exposed to UV light (365 nm) to detect fluorescence, which is indicative of *E. coli* [15].

Heterotrophic bacteria (HB) counts were conducted according to the Brazilian Pharmacopoeia [16]. In compliance with current Brazilian legislation (Ordinance GM/MS N^o^. 888/2021), total coliform and *E. coli* testing was applied as the mandatory baseline screening. Conversely, since HB counts are no longer legally mandatory under this framework, HB analysis was performed as a complementary surveillance tool, with volume of 100 mL of each water sample filtered through a sterile cellulose ester membrane (0.45 µm, 47 mm; Merck Millipore, Germany). The membranes were transferred to Plate Count Agar (PCA) plates and incubated at 35 ± 0.5 °C for 48 h. Colony-forming units (CFU) were counted, and the results were expressed as CFU per mL. A sterility control was carried out by filtering 100 mL of sterile saline, which was subsequently incubated under identical conditions.

### Bacterial isolation and characterisation

Bacterial colonies from samples positive for coliforms or HB growth were isolated by streaking on Tryptone Soy Agar (TSA) plates. After purification, the isolates were Gram-stained and cryopreserved at −20 °C in Tryptone Soy Broth (TSB) with 15% glycerol for future analysis.

Presumptive identification was initially performed through classical biochemical tests, following the guidelines of Mac Faddin [17]. For Gram-negative bacilli, the Bactray® system (Laborclin, Brazil) was used, with kits I and II for oxidase-negative isolates and kit III for oxidase-positive isolates. For the remaining isolates, the online software ABIS 7 (Advanced Bacterial Identification Software) was used. Presumptive *P. aeruginosa* isolates were confirmed with the Pseudoalert® system (IDEXX Laboratories, USA).

The isolates were subjected to MALDI-TOF (Matrix-Assisted Laser Desorption/Ionization Time-of-Flight Mass Spectrometry) mass spectrometry on a Bruker Microflex LT/SH instrument for confirmation. For sample preparation, bacterial colonies were suspended in Milli-Q water, followed by washes in absolute ethanol. 70% formic acid and acetonitrile were added to the dried *pellet* before centrifugation. The final protein extract was applied to a MALDI plate and coated with an α-cyano-4-hydroxycinnamic acid (CHCA) matrix [18]. The mass spectra were compared against the internal library of the MALDI-Biotyper 2011 system. Scores ≥ 2.0 were interpreted as reliable species-level identifications, whereas scores between 1.7 and 1.99 were considered indicative of genus-level identification [19].

### Antibiotic susceptibility testing

Antimicrobial susceptibility was determined by the Kirby-Bauer disk diffusion method, following the Clinical and Laboratory Standards Institute (CLSI) guidelines [20]. Bacterial suspensions were adjusted to the 0.5 McFarland scale standard and inoculated onto Mueller–Hinton (MH) agar plates. Antibiotic disks (Oxoid, UK) were selected based on the identified bacterial species. Zones of inhibition were measured after 24 h of incubation at 35 ± 0.5 °C, and the results were interpreted as sensitive, intermediate, or resistant.

### *DNA extraction and ERIC–PCR of *Pseudomonas aeruginosa* isolates*

Genomic DNA was extracted from *P. aeruginosa* colonies using the boiling method adapted from Dashti et al. [21]. The colonies were suspended in 200 µL of sterile Milli-Q water, boiled for 15 min, and centrifuged. The supernatant, containing the genomic DNA, was collected.

ERIC-PCR amplifications were performed in a final volume of 25 µL, containing the primers ERIC-1R (5′-ATG TAA GCT CCT GGG GAT TCA C) and ERIC-2 (5′-AAG TAA GTG ACT GGG GTG AGC G), 50 ng of extracted DNA and a PCR master mix. The amplification program consisted of an initial denaturation at 95 °C for 2 min, followed by 30 cycles of 90 °C for 30 s, 52 °C for 1 min and 65 °C for 8 min, with a final extension at 72 °C for 10 min [22]. Positive controls (NDM-producing isolates recovered from tracheal aspirate and rectal swab of hospitalized patients) and a negative control (NTC, no template DNA) were included in each reaction.

The amplification products were analyzed by electrophoresis in a 1.5% agarose gel, stained with ethidium bromide (1 μg/mL) and visualized under UV light.

### Data analysis

The band patterns generated by ERIC-PCR were analyzed using BioNumerics® software (Applied Maths, Sint-Martens-Latern, Belgium). Dendrograms were constructed using the UPGMA (Unweighted Pair Group Method with Arithmetic Mean) method. Isolates with a DICE coefficient > 0.93 were considered to belong to the same cluster [22]. For comparison purposes, two isolates representing a cluster of NDM-producing *P. aeruginosa* that circulated at HURM during the same collection period were included in the analysis [14].

### Statistical analysis

Differences in the frequency of samples with heterotrophic bacteria (HB) counts above the regulatory threshold (> 500 CFU/mL) were evaluated using the Chi-square test of independence (χ^2^). Comparisons were performed between samples collected from ICU taps and water tanks and those obtained from the remaining hospital sectors. Statistical significance was established at *p* < 0.05. All analyses were performed using Statistica® version 10.0 (StatSoft Inc., Tulsa, OK, USA).

Additionally, the chronological uniformity of the monthly sampling workflow was evaluated using the coefficient of variation (CV), with lower values indicating greater temporal consistency of sample collection.

## Results

### Assessment of microbiological water quality

A total of 352 water samples were analyzed. The execution of the sampling workflow demonstrated high chronological uniformity, with a mean of 42.17 samples/month (*CV* = 1.78%) for the presence/absence screening and a mean of 16.50 samples/month (*CV* = 3.32%) for the heterotrophic bacteria surveillance, statistically validating the temporal homogeneity of the experimental design. The detection of total coliforms and *E. coli* was performed on 253 of these samples, of which only one (0.4%) tested positive for total coliforms, and none showed the presence of *E. coli*.

HB counting was performed on 99 samples. Of these, 14 samples (14.1%) had counts higher than the reference value of 500 CFU/mL, as established by the previous legislation on water potability, Consolidation Ordinance N^o^. 5 of 2017 [23]. The distribution of results by collection point is detailed in Table 1. Although elevated counts (> 500 CFU/mL) were numerically observed in some water tanks and ICU samples, the Chi-square Test of Independence confirmed that this distribution was not statistically different between hospital sectors (χ^2^ = 0.33, *p* = 0.566). This indicates a statistically homogeneous baseline across the sampling network, confirming that the few instances of elevated HB counts were occasional and isolated events rather than a widespread contamination pattern.

**Table 1:** Frequency of samples contaminated with coliforms and with HB count above the established limit, according to the collection point

	Coliforms			Heterotrophic bacteria	
Locations	Samples (N)	Total (n)	*E. coli* (n)	Samples (N)	Count > 500 CFU/mL (n)
Outpatient clinics	6	0	0	0	0
Milk bank	6	0	0	0	0
Central water tank	6	0	0	6	0
Laundry water tank	6	0	0	7	2
Adult ICU water tank	6	0	0	6	1
General ICU water tank	6	0	0	5	1
Pharmacy	1	0	0	0	0
Surgical and obstetric center	20	0	0	0	0
Surgical clinic	6	1	0	0	0
Gynecological and obstetric clinic	6	0	0	0	0
Medical clinic	6	0	0	0	0
Pediatric clinic	7	0	0	0	0
Material and sterilization center	8	0	0	2	2
Endoscopy room	12	0	0	0	0
Kitchen	12	0	0	0	0
Emergency room	18	0	0	0	0
Hemodialysis room	6	0	0	5	0
Adult ICU (1 e 2)	36	0	0	26	4
General ICU (1 e 2)	36	0	0	31	2
Neonatal ICU	12	0	0	0	0
Pediatric ICU (1, 2 e 3)	28	0	0	11	2
Laboratory	3	0	0	0	0
Total	253	1	0	99	14

N: Number of samples tested; n: Number of contaminated samples; ICU: Intensive care units

### Identification and characterisation of bacterial isolates

After screening based on morphological characteristics, 35 of a total of 67 bacterial isolates were selected for biochemical identification, as shown in Supplementary Material, Table S2. The selection criterion focused on colonies with defined morphology and consistent growth, excluding those with an appearance commonly associated with environmental microorganisms. Among the Gram-negative bacilli, species such as *P. aeruginosa*, *Enterobacter cloacae*, and *Stenotrophomonas maltophilia* stood out. Among the Gram-positive isolates, coagulase-negative *Staphylococcus* spp. and *Bacillus* spp. were identified. The Pseudoalert® chromogenic test confirmed the presence of 10 *P. aeruginosa* isolates.

Presumptive identification by biochemical testing was corroborated by the MALDI-TOF MS method, which confirmed the presence of *P. aeruginosa* and other microorganisms, including *Staphylococcus* spp., *Bacillus* spp., and *Pseudarthrobacter* spp. Table 2 details the frequency, distribution, and identification scores of these isolates. Notably, 14 out of the 35 analyzed isolates (40.0%) presented MALDI-TOF scores below 1.7, indicating that they could not be reliably characterized. These unidentified isolates likely correspond to environmental or atypical water-borne bacteria that are missing from or underrepresented in the MALDI-Biotyper system library (2011) [19].

**Table 2:** Indication of MALDI-Biotyper results

ID	Collection points	Month of identification	BioTyper indication	Average score
ISO1	Laundry water tank	May	Unreliable	1.19
ISO2	Adult ICU water tank	May	Unreliable	1.17
ISO3	Adult ICU water tank	May	*Bacillus* spp.	1.85
ISO4	Adult ICU	May	*Staphylococcus warneri*	2.15
ISO5	Adult ICU	May	Unreliable	1.21
ISO6	Material and sterilization center	June	Unreliable	1.25
ISO7	General ICU	June	*Pseudomonas aeruginosa*	2.13
ISO8	General ICU	July	Unreliable	1.20
ISO9	Pediatric ICU	July	*Pseudomonas* spp.	1.98
ISO10	Pediatric ICU	July	*Enterobacter* spp.	1.82
ISO11	Surgical clinic	July	*Enterobacter* spp.	1.71
ISO12	General ICU	August	*Pseudomonas aeruginosa*	2.38
ISO13	General ICU	August	Unreliable	1.49
ISO14	General ICU	August	*Staphylococcus* spp.	1.89
ISO15	Hemodialysis room	August	*Pseudomonas aeruginosa*	2.18
ISO16	Hemodialysis room	August	Unreliable	1.44
ISO17	Pediatric ICU	August	*Pseudomonas* spp.	1.79
ISO18	General ICU water tank	August	Unreliable	1.26
ISO19	Adult ICU	September	Unreliable	1.45
ISO20	Adult ICU	September	Unreliable	1.34
ISO21	General ICU	September	*Pseudomonas aeruginosa*	2.19
ISO22	General ICU	September	*Staphylococcus epidermidis*	2.00
ISO23	Laundry water tank	September	Unreliable	1.29
ISO24	Laundry water tank	September	*Staphylococcus* spp.	1.90
ISO25	Adult ICU	September	Unreliable	1.43
ISO26	General ICU	September	*Staphylococcus epidermidis*	2.04
ISO27	Adult ICU	October	Unreliable	1.32
ISO28	Adult ICU	October	*Pseudomonas aeruginosa*	2.35
ISO29	Adult ICU	October	*Pseudomonas aeruginosa*	2.23
ISO30	Pediatric ICU	October	*Stenotrophomonas maltophilia*	2.25
ISO31	Pediatric ICU	October	*Stenotrophomonas* spp.	1.78
ISO32	Pediatric ICU	October	*Pseudomonas* spp.	1.97
ISO33	Hemodialysis room	October	*Pseudomonas aeruginosa*	2.21
ISO34	Hemodialysis room	October	Unreliable	1.30
ISO35	General ICU water tank	October	*Pseudarthrobacter* spp.	1.72

ID: Identification of the isolate; ICU: Intensive care units

### Antibiotic susceptibility testing

Antimicrobial susceptibility testing revealed that all *Pseudomonas* spp. isolates were susceptible to carbapenems, ceftazidime, and fluoroquinolones, although a high rate of resistance to cefepime (60%) was observed, with an additional 30% of intermediate isolates. *Enterobacter* spp. isolates exhibited a multidrug-resistant profile, with resistance to extended-spectrum β-lactams, aztreonam, ciprofloxacin, chloramphenicol, trimethoprim-sulfamethoxazole, and tobramycin, whereas remained susceptible to imipenem. The complete table containing all susceptibility test results for the various antimicrobials is available in Table S3 of the Supplementary Material. Finally, among *Staphylococcus* spp., 40% presented resistance to penicillin, trimethoprim-sulfamethoxazole, and azithromycin, with universal susceptibility to cefoxitin, which indicates the absence of methicillin resistance, as shown in Table 3.

**Table 3:** Antibiotic susceptibility testing

Gram-negative oxidase-positive bacilli (P. aeruginosa)																	
ID	CAZ	FEP		ATM		MEM		IPM		CIP		LVX		AMI		TOB	
ISO7	26	14R		28		25		27		43		36		25		25	
ISO9	27	14 R		28		26		26		41		36		23		25	
ISO12	26	14 R		28		28		29		42		37		27		26	
ISO15	26	14 R		29		25		26		40		37		23		26	
ISO17	20	13 R		27		25		27		40		33		24		24	
ISO21	25	15I		28		25		25		41		35		25		25	
ISO28	25	16 I		26		25		27		40		37		25		26	
ISO29	28	20		28		21		30		36		34		28		25	
ISO32	26	13 R		28		26		26		43		34		22		25	
ISO33	26	16 I		27		26		27		39		37		25		26	
Gram-negative oxidase-negative bacilli (ISO10, ISO11: Enterobacter spp.; ISO30, ISO31: S. maltophilia)																	
ID	AMP	AMC	ASB	CFZ	CTX	CAZ	FEP	ATM	ETP	MEM	CIP	LVX	TET	C	SUT	GN	TOB
ISO10	0 R	15 R	8 R	0 R	7 R	15 R	0 R	0 R	10 R	16 R	21 R	26	13 I	0 R	0 R	15 I	12 R
ISO11	0 R	17 R	12 R	0 R	9 R	15 R	0 R	0 R	20 I	21 I	21 R	27	12 I	5 R	0 R	11 R	12 R
ISO30	/	/	/	/	/	/	/	/	/	/	/	36	/	/	16	/	/
ISO31	/	/	/	/	/	/	/	/	/	/	/	36	/	/	17	/	/
Gram-positive bacilli & cocci (ISO3, ISO35: Bacillus spp.; ISO4, ISO14, ISO22, ISO24, ISO26: Staphylococcus spp.)																	
ID	PEN	CFO	CIP	TET	DOX	NIT	SUT	GN	AZI	CLI	RIF						
ISO3	0 R	14 R	26	22	27	20	0 R	25	17 R	20 R	15 R						
ISO35	27	32	26	32	38	19	27	31	37	26	36						
ISO4	10 R	28	29	29	34	24	29	26	0 R	25	34						
ISO14	37	36	38	42	42	32	37	35	33	36	41						
ISO22	37	32	22	28	29	25	0 R	26	26	29	37						
ISO24	17 R	32	33	30	33	31	30	28	0 R	30	37						
ISO26	38	35	32	31	36	28	0 R	30	26	32	38						

ID: Identification of the isolate; PEN: Penicillin 10 μg; AMP: Ampicillin 10 μg; AMC: Amoxicillin-clavulanate 30 μg; ASB: Ampicillin-sulbactam 10/10 μg; CFZ: Cefazolin 30 μg; CFO: Cefoxitin 30 μg; CTX: Cefotaxime 30 μg; CAZ: Ceftazidime 30 μg; FEP: Cefepime 30 μg; ATM: Aztreonam 30 μg; ETP: Ertapenem 10 μg; MEM: Meropenem 10 μg; IPM: Imipenem 10 μg; CIP: Ciprofloxacin 5 μg; LVX: Levofloxacin 5 μg; TET: Tetracycline 30 μg; DOX: Doxycycline 30 μg; C: Chloramphenicol 30 μg; NIT: Nitrofurantoin 300 μg; SUT: Trimethoprim-sulfamethoxazole 1,25/23,75 μg; AMI: Amikacin 30 μg; GN: Gentamicin 120 μg; TOB: Tobramycin 10 μg; AZI: Azithromycin 15 μg; CLI: Clindamycin 2 μg; RIF: Rifampin 5 μg; ^R^: Resistant isolate; ^I^: Sensitivity depending on the dose – Intermediate; /: Not tested.

### Genotypic analysis by ERIC-PCR

**Fig. 1:**
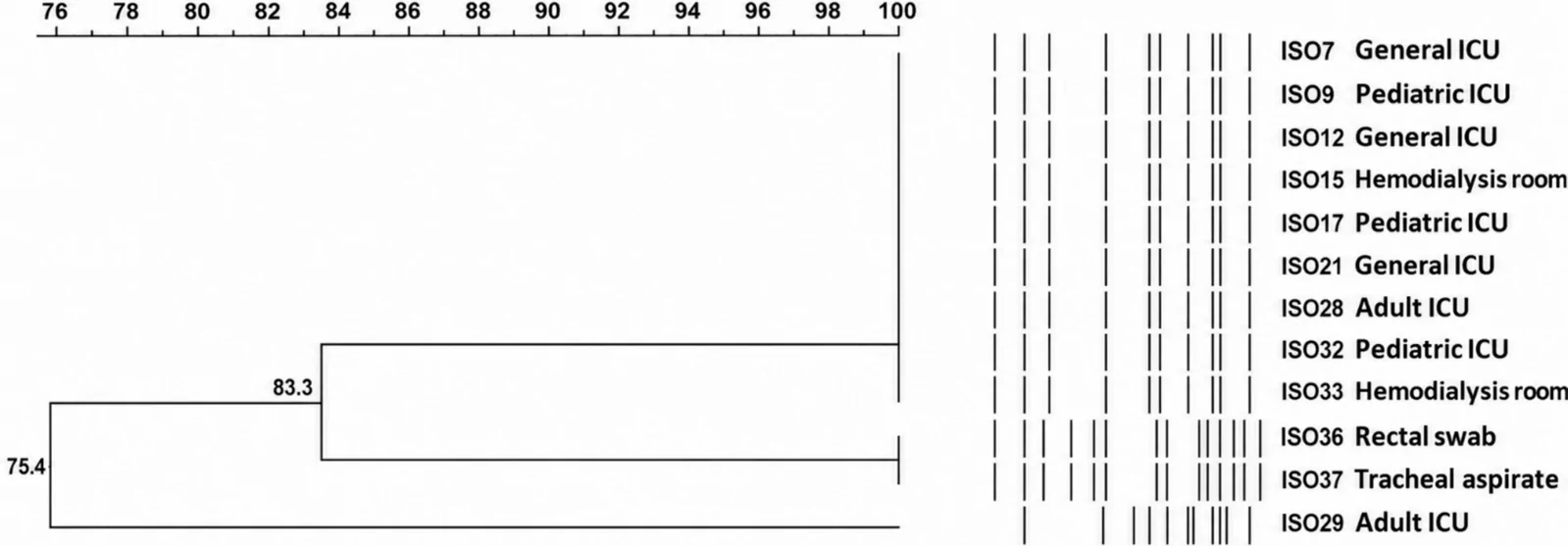
Genetic similarity between environmental *P. aeruginosa* isolates and two representatives of the NDM-producing *P. aeruginosa* cluster isolated from patients (ISO36 and ISO37)

Genotypic evaluation by ERIC-PCR indicated that environmental *P. aeruginosa* isolates presented genetic similarity of less than 0.93 when compared to the two NDM-producing isolates recovered from patients admitted to HURM (ISO36 and 37). This finding demonstrates the absence of a direct clonal relationship between the environmental isolates and the clinical isolates. Despite this, the analysis highlights the genotypic polymorphism of *P. aeruginosa* circulating in the hospital environment. This genetic variability is illustrated by the dendrogram in Fig. 1.

## Discussion

Hospital water distribution systems are increasingly recognized as potential reservoirs of opportunistic microorganisms, complementing the well-established role of hospital wastewater as an environmental source of clinically relevant bacteria and antimicrobial resistance [6, 24, 25]. Within the One Health framework, surveillance of these water systems provides valuable information on the interface between environmental and human health, particularly in healthcare settings where vulnerable patients may be exposed to waterborne opportunistic pathogens [2, 4, 26].

The present six-month surveillance demonstrated that the hospital water distribution network complied with the microbiological potability standards established by Brazilian legislation (Ordinance GM/MS N^o^. 888/2021) [10]. Only one sample (0.4%) tested positive for total coliforms, no sample was positive for *E. coli*, and the positive coliform result did not recur after corrective measures, indicating effective operational control of fecal contamination indicators. Nevertheless, opportunistic bacterial species, including *P. aeruginosa*, *S. maltophilia*, and *Enterobacter* spp., were isolated from treated water, demonstrating that compliance with regulatory potability standards does not necessarily preclude the presence of environmental microorganisms with clinical relevance.

In parallel with these primary potability parameters, elevated HB counts were detected in 17% of water tank samples and 12% of ICU tap samples, no significant differences were observed among hospital sectors (*p* = 0.566). These findings suggest that increased HB counts reflected localized fluctuations rather than widespread microbiological deterioration of the distribution system. Similar observations have been reported in complex hospital plumbing networks, where biofilm formation, water stagnation, and distal outlets may promote intermittent increases in bacterial loads despite overall compliance with drinking water standards [11, 12, 27]. Consequently, although HB counts are no longer mandatory under Brazilian drinking water legislation [10], they remain a useful complementary indicator for environmental surveillance and for identifying critical points requiring preventive maintenance within Water Safety Plans [28].

Our sampling design has specific characteristics that clear up the dataset distribution. In accordance with Brazilian Ordinance GM/MS N^o^. 888/2021 [10], total coliform and *E. coli* surveillance remains legally required, justifying our extensive sampling (*n* = 253) to guarantee baseline potability. On the other hand, because HB counting is no longer mandatory under the current legislation, it was intentionally utilized as a selective operational indicator (*n* = 99). This targeted approach focused exclusively on opportunistic microenvironments with high potential for biofilm development and water stagnation, such as ICUs and water tanks. While this specific sampling prevents the generalization of HB dynamics to the entire structural supply line, it reflects routine environmental monitoring practices and provides a precise diagnostic of the hospital's most vulnerable risk zones. Furthermore, the low frequency of positive baseline events and the small sample sizes in specific sub-sectors represent an intrinsic statistical limitation, restricting advanced multi-variable modeling; however, this distribution accurately reflects the strict operational control of the facility. Additionally, although limited regarding long-term macro-seasonal variations, this six-month continuous window represents a robust operational framework to evaluate immediate bacterial persistence, proving that specific opportunistic taxa can withstand routine distribution treatments and colonize localized plumbing niches across consecutive months.

Although current Brazilian legislation does not establish mandatory limits for HB counts or *P. aeruginosa* research [10], monitoring of these and other parameters is encouraged as part of Water Safety Plans for a more complete risk assessment [28]. This complementary approach is crucial, as a hidden risk may persist for immunocompromised patients even when basic standards are met [29]. The presence of opportunistic pathogens from the ESKAPE group, such as *P. aeruginosa* and *Enterobacter* spp., frequently associated with HAIs, highlights the need to identify microbial reservoirs in the hospital environment, which can facilitate the transmission of bacteria through direct contact, ingestion, aspiration, water aerosolization or via the hands of healthcare staff [30–33].

A substantial proportion of the environmental isolates (40.0%) yielded MALDI-TOF MS scores below the threshold for reliable identification. This finding highlights an important limitation of commercial mass spectrometry databases, particularly older libraries that were developed predominantly from clinical isolates and therefore underrepresent environmental microorganisms [19, 34–37]. Consequently, atypical aquatic bacteria may produce low-confidence identifications despite successful culture recovery. Although 16S rRNA gene sequencing would have provided additional taxonomic resolution, its systematic application was not feasible because of institutional budgetary and logistical constraints. Importantly, all isolates identified with high-confidence MALDI-TOF scores (≥ 2.0) showed complete agreement with conventional biochemical identification, whereas both methodologies demonstrated similar limitations for atypical environmental isolates. Therefore, these unidentified isolates do not compromise the principal conclusions of this study but rather emphasize the complexity and diversity of the microbiota inhabiting hospital water distribution systems and the need for continued improvement of identification databases for environmental surveillance.

The antimicrobial susceptibility profiles varied according to bacterial genus. *P. aeruginosa* isolates remained susceptible to clinically relevant agents, including meropenem, imipenem, ceftazidime, and ciprofloxacin, whereas resistance was mainly observed against cefepime (60%). This susceptibility profile is consistent with findings reported by Lordelo et al. [33], who also described non-multidrug-resistant *P. aeruginosa* isolates recovered from hospital environments, although it contrasts with reports describing multidrug-resistant strains colonizing hospital water systems and plumbing networks [38–41]. In contrast, multidrug resistance was observed exclusively among *Enterobacter* spp. isolates, which exhibited resistance to extended-spectrum β-lactams, third- and fourth-generation cephalosporins, aztreonam, and fluoroquinolones while remaining susceptible to imipenem. Similar resistance patterns have been described in environmental *Enterobacteriaceae* recovered from healthcare facilities [42], reinforcing the importance of monitoring hospital water systems as potential environmental reservoirs of clinically relevant resistant bacteria.

Genotypic analysis by ERIC-PCR confirmed a similarity coefficient below 0.93, identifying no direct clonal relationship between environmental *P. aeruginosa* water isolates and NDM-producing strains recovered from patients. This genetic independence demonstrates that water was not the active source or vehicle for the dissemination of these patient infections during the monitored period [14]. While historical literature, such as studies by Sukhum et al. [38] and Debast et al. [40], highlights successful molecular linkage between plumbing systems and clinical outbreaks via access points, our data yielded no genetic evidence of transmission. These findings indicate that the environmental and clinical isolates represented independent bacterial populations during the surveillance period and suggest that the hospital water network was not an active source of dissemination of the NDM-producing strains investigated. Nevertheless, the detection of opportunistic bacteria in treated water supports the continued microbiological monitoring of hospital water systems as part of infection prevention strategies.

From a One Health perspective, these findings highlight the importance of integrating environmental surveillance into hospital infection prevention programs. Although no direct environmental-to-clinical transmission was demonstrated, treated water remains an important ecological niche where opportunistic microorganisms and antimicrobial resistance traits may persist. Continuous monitoring of hospital water distribution systems can therefore function as an early-warning strategy, complementing conventional clinical surveillance and supporting the implementation of preventive measures before environmental microorganisms become established in high-risk patient areas. This approach is particularly relevant in Brazil, where environmental investigations have focused predominantly on hospital wastewater, whereas studies evaluating the microbiological quality of internal treated water distribution systems remain limited [6, 43–46].

## Supplementary Information

Below is the link to the electronic supplementary material.

Supplementary file1 (DOCX 56 KB)
