# Supplementary material for: One Health in hospital settings: the role of water as an invisible reservoir of opportunistic pathogens
Source: Braz J Microbiol. 2026 Sep 4;57(1):257. doi: 10.1007/s42770-026-02078-5 (PMC13545182; doi:10.1007/s42770-026-02078-5)
Supplement: Supplementary file 1 [file 42770_2026_2078_MOESM1_ESM.docx]

**Supplementary material**

**Table S1**. Monthly distribution of hospital water samples collected for presence/absence testing (total coliforms/*E. coli*) and heterotrophic bacteria (HB) counts over the six-month monitoring period.

| **Locations** | **May** | | **June** | | **July** | | **August** | | **September** | | **October** | | **Total Samples** |
| --- | --- | --- | --- | --- | --- | --- | --- | --- | --- | --- | --- | --- | --- |
|  | **P/A** | **HB** | **P/A** | **HB** | **P/A** | **HB** | **P/A** | **HB** | **P/A** | **HB** | **P/A** | **HB** |  |
| Outpatient clinics | 1 | 0 | 1 | 0 | 1 | 0 | 1 | 0 | 1 | 0 | 1 | 0 | 6 |
| Milk bank | 1 | 0 | 1 | 0 | 1 | 0 | 1 | 0 | 1 | 0 | 1 | 0 | 6 |
| Central water tank | 1 | 1 | 1 | 1 | 1 | 1 | 1 | 1 | 1 | 1 | 1 | 1 | 12 |
| Laundry water tank | 1 | 1 | 1 | 1 | 1 | 1 | 1 | 1 | 1 | 2 | 1 | 1 | 13 |
| Adult ICU water tank | 1 | 1 | 1 | 1 | 1 | 1 | 1 | 1 | 1 | 1 | 1 | 1 | 12 |
| General ICU water tank | 1 | 1 | 1 | 1 | 1 | 1 | 1 | 1 | 1 | 1 | 1 | 0 | 11 |
| Pharmacy | 0 | 0 | 0 | 0 | 0 | 0 | 1 | 0 | 0 | 0 | 0 | 0 | 1 |
| Surgical/obstetric center | 4 | 0 | 3 | 0 | 3 | 0 | 3 | 0 | 4 | 0 | 3 | 0 | 20 |
| Surgical clinic | 1 | 0 | 1 | 0 | 1 | 0 | 1 | 0 | 1 | 0 | 1 | 0 | 6 |
| Gynecological/obstetric clinic | 1 | 0 | 1 | 0 | 1 | 0 | 1 | 0 | 1 | 0 | 1 | 0 | 6 |
| Medical clinic | 1 | 0 | 1 | 0 | 1 | 0 | 1 | 0 | 1 | 0 | 1 | 0 | 6 |
| Pediatric clinic | 2 | 0 | 1 | 0 | 1 | 0 | 1 | 0 | 1 | 0 | 1 | 0 | 7 |
| Material/sterilization center | 1 | 0 | 2 | 2 | 1 | 0 | 1 | 0 | 1 | 0 | 2 | 0 | 10 |
| Endoscopy room | 2 | 0 | 2 | 0 | 2 | 0 | 2 | 0 | 2 | 0 | 2 | 0 | 12 |
| Kitchen | 2 | 0 | 2 | 0 | 2 | 0 | 2 | 0 | 2 | 0 | 2 | 0 | 12 |
| Emergency room | 3 | 0 | 3 | 0 | 3 | 0 | 3 | 0 | 3 | 0 | 3 | 0 | 18 |
| Hemodialysis room | 1 | 1 | 1 | 0 | 1 | 1 | 1 | 1 | 1 | 1 | 1 | 1 | 11 |
| Adult ICU (1 & 2) | 6 | 4 | 6 | 4 | 6 | 4 | 6 | 4 | 6 | 5 | 6 | 5 | 62 |
| General ICU (1 & 2) | 6 | 5 | 6 | 6 | 6 | 5 | 6 | 5 | 6 | 5 | 6 | 5 | 65 |
| Neonatal ICU | 2 | 0 | 2 | 0 | 2 | 0 | 2 | 0 | 2 | 0 | 2 | 0 | 12 |
| Pediatric ICU (1, 2 & 3) | 5 | 2 | 4 | 1 | 5 | 2 | 5 | 2 | 4 | 1 | 5 | 3 | 39 |
| Laboratory | 0 | 0 | 1 | 0 | 1 | 0 | 1 | 0 | 0 | 0 | 0 | 0 | 3 |
| Total (Per Month) | 43 | 16 | 4 | 17 | 42 | 16 | 43 | 16 | 41 | 17 | 42 | 17 | 352 |

Note: P/A = Presence/Absence total coliforms/*E. coli* testing; HB = Heterotrophic bacteria plate count.

**Table S2** Presumptive identification of isolates from water samples

| **ID** | **Gram** | **ESP** | **O_2_R** | **CAT** | **OXI** | **MOT** | **CET** | **ACE** | **MAL** | **CIT** | **MLT** | **ESC** | **URE** | **IND** | **ONG** | **ADH** | **LDC** | **ODC** | **ARG** | **H_2_S** | **GAS** | **MRT** | **VPT** | **PPD** | **G 45** | **G 15** | **COA** | **DNA** | **SAT** | **GLU** | **LAC** | **MLT** | **RAM** | **ADO** | **SAL** | **ARA** | **INO** | **SOR** | **SAC** | **MAN** | **RAF** | **Probable microorganism** |
| --- | --- | --- | --- | --- | --- | --- | --- | --- | --- | --- | --- | --- | --- | --- | --- | --- | --- | --- | --- | --- | --- | --- | --- | --- | --- | --- | --- | --- | --- | --- | --- | --- | --- | --- | --- | --- | --- | --- | --- | --- | --- | --- |
| ISO  1 | GNC | / | fac | + | + | - | / | / | - | - | / | / | / | - | / | / | - | / | / | - | - | - | - | - | - | - | - | - | - | - | - | - | - | / | / | / | / | / | - | - | / | Unidentified |
| ISO  2 | GNB | / | / | / | - | / | / | / | + | + | / | / | - | - | + | - | - | + | / | - | / | / | - | - | / | / | / | / | / | / | / | / | + | - | - | + | - | - | + | + | + | Unidentified |
| ISO  3 | GPB | + | fac | + | - | + | / | / | / | / | / | / | / | - | / | / | / | / | / | - | - | / | + | - | / | / | / | / | / | + | - | - | - | / | / | / | / | / | - | - | / | *Bacillus* spp. |
| ISO  4 | GPC | / | fac | + | - | - | / | / | / | / | / | / | / | / | / | / | / | / | / | - | / | / | + | / | + | - | - | - | + | + | - | + | - | / | / | / | / | / | + | - | / | Coagulase-negative *Staphylococcus* spp. |
| ISO  5 | GPC | / | fac | + | - | - | / | / | / | / | / | / | / | / | / | / | / | / | / | - | / | / | + | / | + | - | - | - | + | + | + | - | - | / | / | / | / | / | + | - | / | Coagulase-negative *Staphylococcus* spp. |
| ISO  6 | GNB | / | / | / | + | / | - | - | + | + | - | - | - | - | / | / | / | / | + | / | / | / | / | / | / | / | / | / | / | / | / | - | / | / | / | / | / | / | / | / | / | Unidentified |
| ISO  7 | GNB | / | / | / | + | / | + | + | + | + | - | + | + | - | / | / | / | / | + | / | / | / | / | / | / | / | / | / | / | / | / | - | / | / | / | / | / | / | / | / | / | *Pseudomonas aeruginosa* |
| ISO  8 | GNB | / | / | / | - | / | / | / | + | + | / | / | - | - | + | - | - | + | / | - | / | / | - | - | / | / | / | / | / | / | / | / | + | - | - | + | - | - | + | + | + | Unidentified |
| ISO  9 | GNB | / | / | / | + | / | + | + | + | + | - | + | + | - | / | / | / | / | + | / | / | / | / | / | / | / | / | / | / | / | / | - | / | / | / | / | / | / | / | / | / | *Pseudomonas aeruginosa* |
| ISO  10 | GNB | / | / | / | - | / | / | / | + | + | / | / | - | - | + | + | - | + | / | - | / | / | + | - | / | / | / | / | / | / | / | / | + | - | - | + | - | - | + | + | + | *Enterobacter cloacae* |
| ISO  11 | GNB | / | / | / | - | / | / | / | + | + | / | / | - | - | + | + | - | + | / | - | / | / | + | - | / | / | / | / | / | / | / | / | + | - | - | + | - | + | + | + | + | *Enterobacter cloacae* |
| ISO  12 | GNB | / | / | / | + | / | + | + | + | + | - | + | + | - | / | / | / | / | + | / | / | / | / | / | / | / | / | / | / | / | / | - | / | / | / | / | / | / | / | / | / | *Pseudomonas aeruginosa* |
| ISO  13 | GNB | / | / | / | **+** | / | - | - | - | - | + | - | - | - | / | / | / | / | + | / | / | / | / | / | / | / | / | / | / | / | / | + | / | / | / | / | / | / | / | / | / | Unidentified |
| ISO  14 | GPC | / | fac | + | - | + | / | / | / | / | / | / | / | / | / | / | / | / | / | - | / | / | + | / | + | - | - | - | + | + | + | - | - | / | / | / | / | / | + | - | / | Coagulase-negative *Staphylococcus* spp. |
| ISO  15 | GNB | / | / | / | + | / | + | + | + | + | - | + | + | - | / | / | / | / | + | / | / | / | / | / | / | / | / | / | / | / | / | - | / | / | / | / | / | / | / | / | / | *Pseudomonas aeruginosa* |
| ISO  16 | GNB | / | / | / | + | / | + | - | + | + | - | - | - | - | / | / | / | / | + | / | / | / | / | / | / | / | / | / | / | / | / | - | / | / | / | / | / | / | / | / | / | Unidentified |
| ISO  17 | GNB | / | / | / | + | / | + | + | + | + | - | + | + | - | / | / | / | / | + | / | / | / | / | / | / | / | / | / | / | / | / | - | / | / | / | / | / | / | / | / | / | *Pseudomonas aeruginosa* |
| ISO  18 | GNB | / | / | / | - | / | / | / | - | - | / | / | - | - | - | - | - | + | / | - | / | / | - | - | / | / | / | / | / | / | / | / | + | - | - | - | - | - | - | - | - | Unidentified |
| ISO  19 | GNB | / | / | / | - | / | / | / | + | + | / | / | - | - | + | - | - | + | / | - | / | / | - | - | / | / | / | / | / | / | / | / | + | - | - | + | - | - | + | + | + | Unidentified |
| ISO  20 | GNB | / | / | / | + | / | + | - | + | + | - | - | - | - | / | / | / | / | + | / | / | / | / | / | / | / | / | / | / | / | / | - | / | / | / | / | / | / | / | / | / | Unidentified |
| ISO  21 | GNB | / | / | / | + | / | + | + | + | + | - | + | + | - | / | / | / | / | + | / | / | / | / | / | / | / | / | / | / | / | / | - | / | / | / | / | / | / | / | / | / | *Pseudomonas aeruginosa* |
| ISO  22 | GPC | / | fac | + | - | - | / | / | / | / | / | / | / | / | / | / | / | / | / | - | / | / | + | / | + | - | - | - | + | + | + | + | + | / | / | / | / | / | + | - | / | Coagulase-negative *Staphylococcus* spp. |
| ISO  23 | GNC | / | fac | + | + | - | / | / | + | + | / | / | / | - | / | / | - | / | / | - | + | - | + | - | + | - | - | - | - | + | - | + | + | / | / | / | / | / | - | + | / | Unidentified |
| ISO  24 | GPC | / | fac | + | - | - | / | / | / | / | / | / | / | / | / | / | / | / | / | - | / | / | + | / | + | - | - | - | + | + | - | + | - | / | / | / | / | / | + | - | / | Coagulase-negative *Staphylococcus* spp. |
| ISO  25 | GNB | / | / | / | + | / | - | + | - | - | - | + | + | - | / | / | / | / | + | / | / | / | / | / | / | / | / | / | / | / | / | - | / | / | / | / | / | / | / | / | / | Unidentified |
| ISO  26 | GPC | / | fac | + | - | - | / | / | / | / | / | / | / | / | / | / | / | / | / | - | / | / | + | / | + | - | - | - | + | + | + | + | - | / | / | / | / | / | + | - | / | Coagulase-negative *Staphylococcus* spp. |
| ISO  27 | GNB | / | / | / | + | / | - | - | + | + | - | - | - | - | / | / | / | / | + | / | / | / | / | / | / | / | / | / | / | / | / | - | / | / | / | / | / | / | / | / | / | Unidentified |
| ISO  28 | GNB | / | / | / | + | / | + | + | + | + | - | + | + | - | / | / | / | / | + | / | / | / | / | / | / | / | / | / | / | / | / | - | / | / | / | / | / | / | / | / | / | *Pseudomonas aeruginosa* |
| ISO  29 | GNB | / | / | / | + | / | + | + | + | + | - | + | + | - | / | / | / | / | + | / | / | / | / | / | / | / | / | / | / | / | / | - | / | / | / | / | / | / | / | / | / | *Pseudomonas aeruginosa* |
| ISO  30 | GNB | / | / | / | - | / | / | / | + | - | / | / | - | - | - | + | + | + | / | - | / | / | - | - | / | / | / | / | / | / | / | / | - | - | - | - | - | - | - | - | - | *Stenotrophomonas maltophilia* |
| ISO  31 | GNB | / | / | / | - | / | / | / | + | - | / | / | - | - | - | + | + | + | / | - | / | / | - | - | / | / | / | / | / | / | / | / | - | - | - | - | - | - | - | - | - | *Stenotrophomonas maltophilia* |
| ISO  32 | GNB | / | / | / | + | / | + | + | + | + | - | + | + | - | / | / | / | / | + | / | / | / | / | / | / | / | / | / | / | / | / | - | / | / | / | / | / | / | / | / | / | *Pseudomonas aeruginosa* |
| ISO  33 | GNB | / | / | / | + | / | + | + | + | + | - | + | + | - | / | / | / | / | + | / | / | / | / | / | / | / | / | / | / | / | / | - | / | / | / | / | / | / | / | / | / | *Pseudomonas aeruginosa* |
| ISO  34 | GNB | / | / | / | - | / | / | / | - | - | / | / | - | - | - | - | - | + | / | - | / | / | - | - | / | / | / | / | / | / | / | / | - | - | - | - | - | - | - | - | - | Unidentified |
| ISO  35 | GPB | - | + | + | - | - | / | / | / | / | / | / | / | - | / | / | / | / | / | - | / | / | - | / | - | - | - | - | - | - | - | - | - | / | / | / | / | / | - | - | / | Unidentified |

ID: Identification of the isolate; ESP: Endospore formation; O₂R: O_2_ requirement; CAT: Catalase; OXI: Oxidase; MOT: Motility; CET: Growth on cetrimide agar; ACE: Growth on acetamide; MAL: Utilization of malonate; CIT: Utilization of citrate; ESC: Degradation of esculin; URE: Degradation of urea; IND: Production of indole; ONG: Production of β-galactosidase; ADH: Activity of arginine dihydrolase; LDC: Decarboxylation of lysine; ODC: Decarboxylation of ornithine; ARG: Decarboxylation of arginine; H_2_S: Production of H_2_S; MRT: Methyl red test; VPT: Voges–Proskauer test; PPD: Production of phenylalanine deaminase; G45: Growth at 45 °C; G15: Growth at 15 °C; COA: Tube agglutination test; DNA: Degradation of DNA; SAT: Salt tolerance, growth in 10% NaCl; GLU: Acid produced from glucose; LAC: Acid produced from lactose; MLT: Acid produced from maltose; RAM: Acid produced from rhamnose; ADO: Acid produced from adonitol; SAL: Acid produced from salicin; ARA: Acid produced from arabinose; INO: Acid produced from inositol; SOR: Acid produced from sorbitol; SAC: Acid produced from sucrose; MAN: Acid produced from mannitol; RAF: Acid produced from raffinose; GNC: Gram-negative cocci; GPC: Gram-positive cocci; GPB: Gram-positive bacilli; GNB: Gram-negative bacilli; fac: facultative; /: Not tested.

| **Gram-negative oxidase-negative bacilli** | | | | | | | | | | | | | | | | | | | | | | |
| --- | --- | --- | --- | --- | --- | --- | --- | --- | --- | --- | --- | --- | --- | --- | --- | --- | --- | --- | --- | --- | --- | --- |
| **ID** | **A**  **M**  **P** | **A**  **M**  **C** | **A**  **S**  **B** | **C**  **F**  **Z** | **C**  **F**  **O** | **C**  **T**  **X** | **C**  **A**  **Z** | **F**  **E**  **P** | **A**  **T**  **M** | **E**  **T**  **P** | **M**  **E**  **M** | **I**  **PM** | **C**  **I**  **P** | **L**  **V**  **X** | **T**  **E**  **T** | **D**  **O**  **X** | **C** | **N**  **I**  **T** | **S**  **U**  **T** | **A**  **M**  **I** | **G**  **N** | **T**  **O**  **B** |
| ISO10 | 0  ^R^ | 15  ^R^ | 8  ^R^ | 0  ^R^ | 24 | 7  ^R^ | 15  ^R^ | 0  ^R^ | 0  ^R^ | 10  ^R^ | 16  ^R^ | 27 | 21  ^R^ | 26 | 13  ^I^ | 18 | 0  ^R^ | 21 | 0  ^R^ | 21 | 15  ^I^ | 12  ^R^ |
| ISO11 | 0  ^R^ | 17  ^R^ | 12  ^R^ | 0  ^R^ | 24 | 9  ^R^ | 15  ^R^ | 0  ^R^ | 0  ^R^ | 20  ^I^ | 21  ^I^ | 29 | 21  ^R^ | 27 | 12  ^I^ | 17 | 5  ^R^ | 22 | 0  ^R^ | 22 | 11  ^R^ | 12  ^R^ |

**Table S3** Antibiotic susceptibility testing for *Enterobacter* spp. isolates.

ID: Identification of the isolate; AMP: Ampicillin 10 μg; AMC: Amoxicillin-clavulanate 30 μg; ASB: Ampicillin-sulbactam 10/10 μg; CFZ: Cefazolin 30 μg; CFO: Cefoxitin 30 μg; CTX: Cefotaxime 30 μg; CAZ: Ceftazidime 30 μg; FEP: Cefepime 30 μg; ATM: Aztreonam 30 μg; ETP: Ertapenem 10 μg; MEM: Meropenem 10 μg; IPM: Imipenem 10 μg; CIP: Ciprofloxacin 5 μg; LVX: Levofloxacin 5 μg; TET: Tetracycline 30 μg; DOX: Doxycycline 30 μg; C: Chloramphenicol 30 μg; NIT: Nitrofurantoin 300 μg; SUT: Trimethoprim-sulfamethoxazole 1,25/23,75 μg; AMI: Amikacin 30 μg; GN: Gentamicin 120 μg; TOB: Tobramycin 10 μg; R: Resistant isolate; I: Sensitivity depending on the dose – Intermediate;
